# Pooled safety analysis of two phase 3 studies investigating trifluridine/tipiracil plus bevacizumab in patients with metastatic colorectal cancer

**DOI:** 10.3389/fonc.2024.1506075

**Published:** 2025-01-16

**Authors:** Julien Taieb, Marwan Fakih, Gabor Liposits, Gerald W. Prager, Eric Van Cutsem, Fortunato Ciardiello, Nadia Amellal, Elizabeth Calleja, Mei Liu, Lucas Roby, Josep Tabernero, Thierry André

**Affiliations:** ^1^ Hôpital Européen Georges-Pompidou, University Paris-Cité (Paris Descartes), SIRC CARPEM, Paris, France; ^2^ Department of Medical Oncology and Therapeutics Research, City of Hope Comprehensive Cancer Center, Duarte, CA, United States; ^3^ Department of Oncology, Gødstrup Hospital, Herning, Denmark; ^4^ Department of Medicine I, Medical University Vienna, Vienna, Austria; ^5^ Digestive Oncology, University Hospitals Gasthuisberg Leuven and University of Leuven, Leuven, Belgium; ^6^ Department of Precision Medicine, University of Campania Liuigi Vanvitelli, Naples, Italy; ^7^ Servier International Research Institute, Suresnes, France; ^8^ Taiho Oncology, Inc., Princeton, NJ, United States; ^9^ Vall d’Hebron Hospital Campus, Vall d’Hebron Institute of Oncology (VHIO), Barcelona, Spain; ^10^ Sorbonne University and Saint-Antoine Hospital, APHP, Paris, France

**Keywords:** clinical trial, first-line, SOLSTICE, SUNLIGHT, third-line

## Abstract

**Background:**

Trifluridine/tipiracil (FTD/TPI) is approved as monotherapy and in combination with bevacizumab for the treatment of patients with refractory metastatic colorectal cancer (mCRC). FTD/TPI plus bevacizumab showed good tolerability in the phase 3 SOLSTICE (first-line) and SUNLIGHT (later-line) trials. This pooled analysis was performed to further characterize the safety of FTD/TPI plus bevacizumab and to compare safety in untreated and previously treated patients with mCRC.

**Methods:**

Patients must have received at least one dose of FTD/TPI plus bevacizumab in SOLSTICE (NCT03869892) or SUNLIGHT (NCT04737187). Treatment-emergent adverse events (TEAEs) in SOLSTICE and SUNLIGHT were graded per Common Terminology Criteria for Adverse Events versions 4.03 and 5.0, respectively. Times to onset/resolution of grade ≥3 hematologic TEAEs were assessed using Kaplan–Meier methodology. Treatment-related adverse events (TRAEs) were analyzed by age and Eastern Cooperative Oncology Group performance status (ECOG PS).

**Results:**

The pooled safety population comprised 669 patients (SOLSTICE, n = 423; and SUNLIGHT, n = 246). Grade ≥3 TEAEs were reported more frequently in SOLSTICE than in SUNLIGHT (86.8% vs. 72.4%), the most common being neutropenia and anemia. Overall, granulocyte colony-stimulating factor was used in 30.6% of patients. Median time to resolution of grade ≥3 hematologic adverse events/neutropenia to grade ≤2 was 8 days. Grade ≥3 TRAEs were more frequent in patients aged ≥75 years and those with an ECOG PS of 0 versus 1 or 2.

**Conclusions:**

FTD/TPI plus bevacizumab showed a consistent and manageable safety profile across first- and later-line mCRC treatment, including in vulnerable patients. Hematologic TEAEs were mostly reversible with appropriate management.

## 1 Introduction

Trifluridine/tipiracil (FTD/TPI) is an oral combination of trifluridine (FTD), a cytotoxic thymidine–based nucleoside analog, and tipiracil hydrochloride, a thymidine phosphorylase inhibitor that prevents degradation of and improves systemic exposure to FTD (1). FTD/TPI is approved for the third- or later-line treatment of metastatic colorectal cancer (mCRC), either as monotherapy or in combination with bevacizumab, based on results from the phase 3 RECOURSE and SUNLIGHT trials, respectively (2–4), with the combination recommended in the National Comprehensive Cancer Network (NCCN) guidelines and the mCRC Living Guidelines of the European Society for Medical Oncology for patients previously treated with fluoropyrimidines, oxaliplatin, irinotecan, and biologics (5, 6).

The rationale for combining FTD/TPI with bevacizumab is based on their independent mechanisms of action, along with preclinical evidence showing that bevacizumab increases FTD accumulation in tumor cell DNA (7, 8). Clinically, FTD/TPI has shown efficacy and acceptable tolerability in combination with bevacizumab in first-line (SOLSTICE) and later-line (SUNLIGHT) mCRC, as well as in several phase 2 trials (4, 9–12). In the phase 3 SOLSTICE trial, conducted in patients who were not candidates for intensive therapy, the primary endpoint was not met. First-line FTD/TPI plus bevacizumab showed similar progression-free survival to capecitabine plus bevacizumab [median, 9.4 vs. 9.3 months; hazard ratio (HR), 0.87; 95% confidence interval (CI), 0.75–1.02; p = 0.0464] (9); median overall survival was also similar between treatment arms (19.7 vs. 18.6 months; HR, 1.06; 95% CI, 0.90–1.25) (13). However, in SUNLIGHT, treatment with FTD/TPI plus bevacizumab resulted in significantly longer overall survival (median, 10.8 vs. 7.5 months; HR, 0.61; 95% CI, 0.49–0.77; p < 0.001) and progression-free survival (5.6 vs. 2.4 months; HR, 0.44; 95% CI, 0.36–0.54; p < 0.001) than FTD/TPI in patients who had received no more than two prior chemotherapy regimens (4). Safety findings from the SOLSTICE and SUNLIGHT trials showed that treatment-emergent adverse events (TEAEs) with FTD/TPI plus bevacizumab were consistent with the known safety profiles of FTD/TPI and bevacizumab individually, the most common being hematologic toxicities, gastrointestinal adverse events (AEs), fatigue, and hypertension (7, 10). In SOLSTICE, compared with capecitabine plus bevacizumab, first-line FTD/TPI plus bevacizumab was associated with a higher rate of neutropenia but a lower rate of hand-foot syndrome (9). Grade ≥3 neutropenia, but not febrile neutropenia, was more common with FTD/TPI plus bevacizumab than with FTD/TPI alone in SUNLIGHT (4).

The objectives of the current analysis were to further characterize the overall safety of FTD/TPI plus bevacizumab in previously untreated or refractory patients with mCRC and to compare the safety of the combination in first- and later-line patient populations.

## 2 Methods

### 2.1 Patients and study designs

This analysis included data from patients with mCRC who received at least one dose of FTD/TPI plus bevacizumab in SOLSTICE (NCT03869892) or SUNLIGHT (NCT04737187). Full details of the study designs and eligibility criteria have been published previously (4, 9, 14, 15). Both studies were global, open-label, randomized, phase 3 trials that enrolled adult patients with histologically confirmed, unresectable adenocarcinoma of the colon or rectum, known *RAS* mutation status, adequate organ function, and an estimated life expectancy of ≥12 weeks. Eligibility for SOLSTICE required an Eastern Cooperative Oncology Group performance status (ECOG PS) of 0–2, whereas, in SUNLIGHT, an ECOG PS of 0 or 1 was allowed. In SOLSTICE, patients were previously untreated and were not candidates for intensive combination chemotherapy with irinotecan or oxaliplatin per investigator judgment, due to clinical (e.g., ECOG PS, comorbidities, and age >70 years) and/or nonclinical (e.g., low tumor burden and patient preference) conditions. In SUNLIGHT, patients must have received no more than two prior chemotherapy regimens containing fluoropyrimidines, irinotecan, oxaliplatin, a vascular endothelial growth factor inhibitor, and/or (in patients with *RAS* wild-type tumors) an epidermal growth factor receptor inhibitor and have had disease progression or intolerance to the last regimen.

Patients were randomized (1:1) to either FTD/TPI 35 mg/m^2^ orally twice daily on days 1 through 5 and days 8 through 12, plus bevacizumab 5 mg/kg intravenously on days 1 and 15 of each 28-day cycle (SOLSTICE and SUNLIGHT); or capecitabine 1,250 or 1,000 mg/m^2^ orally twice daily on days 1 through 14, plus bevacizumab 7.5 mg/kg intravenously on day 1 of each 21-day cycle (SOLSTICE); or FTD/TPI alone (SUNLIGHT). Treatment continued until disease progression, unacceptable toxicity, or withdrawal of consent.

Both trials were performed in accordance with the principles of the Declaration of Helsinki, good clinical practice, and applicable regulatory requirements. The study protocols were approved by the institutional review board(s) and/or independent ethics committee(s) at each participating center. All enrolled patients provided written informed consent.

### 2.2 Safety assessments

The pooled safety analysis included data collected up to the data cutoff dates of 9 June 2021 for SOLSTICE and 5 July 2022 for SUNLIGHT. AEs were coded using the Medical Dictionary for Regulatory Activities version 25.0 and graded per the National Cancer Institute Common Terminology Criteria for Adverse Events (NCI-CTCAE) version 4.03 (SOLSTICE) or 5.0 (SUNLIGHT). Assessment of hematologic AEs was conducted on the basis of NCI-CTCAE definitions and laboratory parameters. For SOLSTICE, NCI-CTCAE version 5.0 was used for laboratory parameters in the pooled analysis, based on numeric criteria alone, without additional clinical information. AEs were summarized as those occurring from the initiation of treatment administration to 30 days after the last dose. Details of the protocol-defined management of AEs, including dose modifications and supportive care interventions, are provided as **Supplementary Material** (**Supplementary Table 1**).

### 2.3 *Post-hoc* and statistical analysis

All safety data are presented descriptively. No formal hypothesis testing was performed. *Post-hoc* analyses included assessment of the timing of onset and resolution of grade ≥3 hematologic AEs, calculated using Kaplan–Meier methodology, with 95% CIs based on the Greenwood formula. Subgroup analyses of treatment-related AEs (TRAEs) by age and ECOG PS were also conducted.

## 3 Results

### 3.1 Patients and treatment

The pooled safety population comprised 669 patients who received FTD/TPI plus bevacizumab in SOLSTICE (n = 423) and SUNLIGHT (n = 246; **Table 1**). The median age was 69.0 years, 53.8% of patients were men, and 61.4% were enrolled from the European Union. A greater proportion of the SOLSTICE patient population was aged ≥75 years compared with that of the SUNLIGHT population.

**Table 1:** Baseline characteristics of patients receiving FTD/TPI plus bevacizumab in the SOLSTICE, SUNLIGHT, and pooled safety populations.

Characteristic, n (%) unless otherwise specified		SOLSTICE (N = 423)	SUNLIGHT (N = 246)	Total (N = 669)
Median (range) age, years		73.0 (27–93)	62.0 (20–84)	69.0 (20–63)
	<75 years	235 (55.6)	222 (90.2)	457 (68.3)
	≥75 years	188 (44.4)	24 (9.8)	212 (31.7)
Male		238 (56.3)	122 (49.6)	360 (53.8)
Race	White	405 (95.7)	215 (87.4)	620 (92.7)
	Black/African American	2 (0.5)	4 (1.6)	6 (0.9)
	Asian	3 (0.7)	0	3 (0.4)
	Other	11 (2.6)	9 (3.7)	20 (3.0)
	Missing	2 (0.5)	18 (7.3)	20 (3.0)
Geographic region	North America	0	8 (3.3)	8 (1.2)
	European Union	253 (59.8)	158 (64.2)	411 (61.4)
	Rest of the world	170 (40.2)	80 (32.5)	250 (37.4)
ECOG PS	0	97 (22.9)	119 (48.4)	216 (32.3)
	1	246 (58.2)	127 (51.6)	373 (55.8)
	2	80 (18.9)	0	80 (12.0)
Renal function at baseline	Creatinine clearance <60 mL/min	118 (27.9)	30 (12.2)	148 (22.1)
	Creatinine clearance ≥60 mL/min	304 (71.9)	215 (87.4)	519 (77.6)
	Missing	1 (0.2)	1 (0.4)	2 (0.3)
*RAS* mutation status	Wild-type	177 (41.8)	71 (28.9)	248 (37.1)
	Mutant	236 (55.8)	174 (70.7)	410 (61.3)
	Not evaluable	10 (2.4)	1 (0.4)	11 (1.6)
Primary cancer diagnosis (adenocarcinoma)	Colon	328 (77.5)	180 (73.2)	508 (75.9)
	Rectum	95 (22.5)	66 (26.8)	161 (24.1)
Primary tumor localization	Right	129 (30.5)	62 (25.2)	191 (28.6)
	Left	294 (69.5)	184 (74.8)	478 (71.4)
Median (range) time since diagnosis, years		0.3 (0.02–25.0)	2.0 (0.31–15.4)	1.2 (0.02–25.0)
Median (range) time since first metastasis diagnosis, months		1.2 (0.03–41.6)	21.0 (0.62–133.2)	2.4 (0.03–133.2)
Prior surgery		268 (63.4)	156 (63.4)	424 (63.4)
Prior radiotherapy		47 (11.1)	38 (15.4)	85 (12.7)

ECOG PS, Eastern Cooperative Oncology Group performance status.

### 3.2 Overall safety

The median (range) duration of treatment was 8.2 (0.3–24.4) months in SOLSTICE and 5.0 (0.1–18.5) months in SUNLIGHT.

In the pooled safety population, TEAEs were reported by 98.5% of patients (**Table 2**). The most common, regardless of causality, were neutropenia, anemia, nausea, diarrhea, asthenia, fatigue, and decreased appetite. Grade ≥3 TEAEs were reported more frequently in SOLSTICE than in SUNLIGHT (86.8% and 72.4%, respectively). The most common grade ≥3 TEAEs in the pooled safety population were neutropenia (60.2%) and anemia (11.2%; **Table 2**).

**Table 2:** Overall safety summary and most common any-grade/grade ≥3 TEAEs among patients receiving FTD/TPI plus bevacizumab in the SOLSTICE, SUNLIGHT, and pooled safety populations.

Patients with TEAEs, n (%)		SOLSTICE (N = 423)		SUNLIGHT (N = 246)		Total (N = 669)	
Any TEAE		418 (98.8)		241 (98.0)		659 (98.5)	
	Grade ≥3 TEAE	367 (86.8)		178 (72.4)		545 (81.5)	
Treatment-related AE		396 (93.6)		223 (90.7)		619 (92.5)	
	Related to FTD/TPI	391 (92.4)		221 (89.8)		612 (91.5)	
	Related to bevacizumab	239 (56.5)		119 (48.4)		358 (53.5)	
	Grade ≥3 treatment-related TEAE	329 (77.8)		145 (58.9)		474 (70.9)	
SAE		160 (37.8)		60 (24.4)		220 (32.9)	
	Treatment-related SAE	70 (16.5)		12 (4.9)		82 (12.3)	
Fatal AE		33 (7.8)		13 (5.3)		46 (6.9)	
Treatment-related fatal TEAE		5 (1.2)		0		5 (0.7)	
TEAE leading to dose delay		301 (71.2)		174 (70.7)		475 (71.0)	
TEAE leading to dose reduction		107 (25.3)		40 (16.3)		147 (22.0)	
TEAE leading to dose interruption		66 (15.6)		73 (29.7)		139 (20.8)	
TEAE leading to treatment discontinuation		49 (11.6)		36 (14.6)		85 (12.7)	
Most common AEs^a^, n (%)		SOLSTICE (N = 423)		SUNLIGHT (N = 246)		Total (N = 669)	
		Any grade	Grade ≥3	Any grade	Grade ≥3	Any grade	Grade ≥3
Neutropenia		339 (80.1)	281 (66.4)	176 (71.5)	122 (49.6)	515 (77.0)	403 (60.2)
Anemia		188 (44.4)	60 (14.2)	71 (28.9)	15 (6.1)	259 (38.7)	75 (11.2)
Nausea		148 (35.0)	7 (1.7)	91 (37.0)	4 (1.6)	239 (35.7)	11 (1.6)
Diarrhea		154 (36.4)	30 (7.1)	51 (20.7)	2 (0.8)	205 (30.6)	32 (4.8)
Asthenia		95 (22.5)	26 (6.1)	60 (24.4)	10 (4.1)	155 (23.2)	36 (5.4)
Fatigue		101 (23.9)	25 (5.9)	53 (21.5)	3 (1.2)	154 (23.0)	28 (4.2)
Decreased appetite		95 (22.5)	7 (1.7)	50 (20.3)	2 (0.8)	145 (21.7)	9 (1.3)
Thrombocytopenia		81 (19.1)	16 (3.8)	42 (17.1)	7 (2.8)	123 (18.4)	23 (3.4)
Vomiting		68 (16.1)	7 (1.7)	46 (18.7)	2 (0.8)	114 (17.0)	9 (1.3)
Leukopenia		71 (16.8)	23 (5.4)	16 (6.5)	4 (1.6)	87 (13.0)	27 (4.0)
Stomatitis		55 (13.0)	6 (1.4)	27 (11.0)	1 (0.4)	82 (12.3)	7 (1.0)
Hypertension		56 (13.2)	36 (8.5)	25 (10.2)	14 (5.7)	81 (12.1)	50 (7.5)
Abdominal pain		50 (11.8)	7 (1.7)	29 (11.8)	5 (2.0)	79 (11.8)	12 (1.8)
Constipation		51 (12.1)	2 (0.5)	27 (11.0)	0	78 (11.7)	2 (0.3)
Weight decreased		47 (11.1)	2 (0.5)	20 (8.1)	2 (0.8)	67 (10.0)	4 (0.6)
Hypokalemia		18 (4.3)	13 (3.1)	5 (2.0)	1 (0.4)	23 (3.4)	14 (2.1)

AE, adverse event; FTD/TPI, trifluridine and tipiracil; SAE, serious adverse event; TEAE, treatment-emergent adverse event.

a AEs of any grade occurring in ≥10% of patients or grade ≥3 AEs occurring in ≥3% of patients in either study, excluding malignant neoplasm progression.

Most patients (92.5%) had TEAEs that were considered related to the combination of FTD/TPI plus bevacizumab, the most common of which were neutropenia (86.8%), anemia (35.0%), nausea (31.9%), and diarrhea (31.0%) in SOLSTICE and neutropenia (74.0%), nausea (33.3%), anemia (25.2%), and asthenia (19.1%) in SUNLIGHT. Bevacizumab-related hypertension was reported in 8.3% and 7.3% of patients in SOLSTICE and SUNLIGHT, respectively. Cardiac disorders related to bevacizumab were reported in nine (2.1%) patients in SOLSTICE, including cardiac failure in three patients and atrial fibrillation in two patients; no bevacizumab-related cardiac disorders were reported in SUNLIGHT.

Serious AEs (SAEs; 37.8% vs. 24.4%), treatment-related SAEs (16.5% vs. 4.9%), and AEs leading to dose reduction (25.3% vs. 16.3%) were more common in SOLSTICE; AEs leading to dose interruption (15.6% vs. 29.7%) or treatment discontinuation (11.6% vs. 14.6%) were more common in SUNLIGHT. Five (1.2%) patients in SOLSTICE died due to TRAEs [Dieulafoy’s vascular malformation and gastric hemorrhage; urosepsis; pulmonary embolism and pulmonary hemorrhage; chronic cardiac failure; and cardiorespiratory arrest (all n = 1)]; there were no treatment-related deaths in SUNLIGHT.

### 3.3 Analyses of hematologic AEs in the pooled safety population

**Figure 1:**
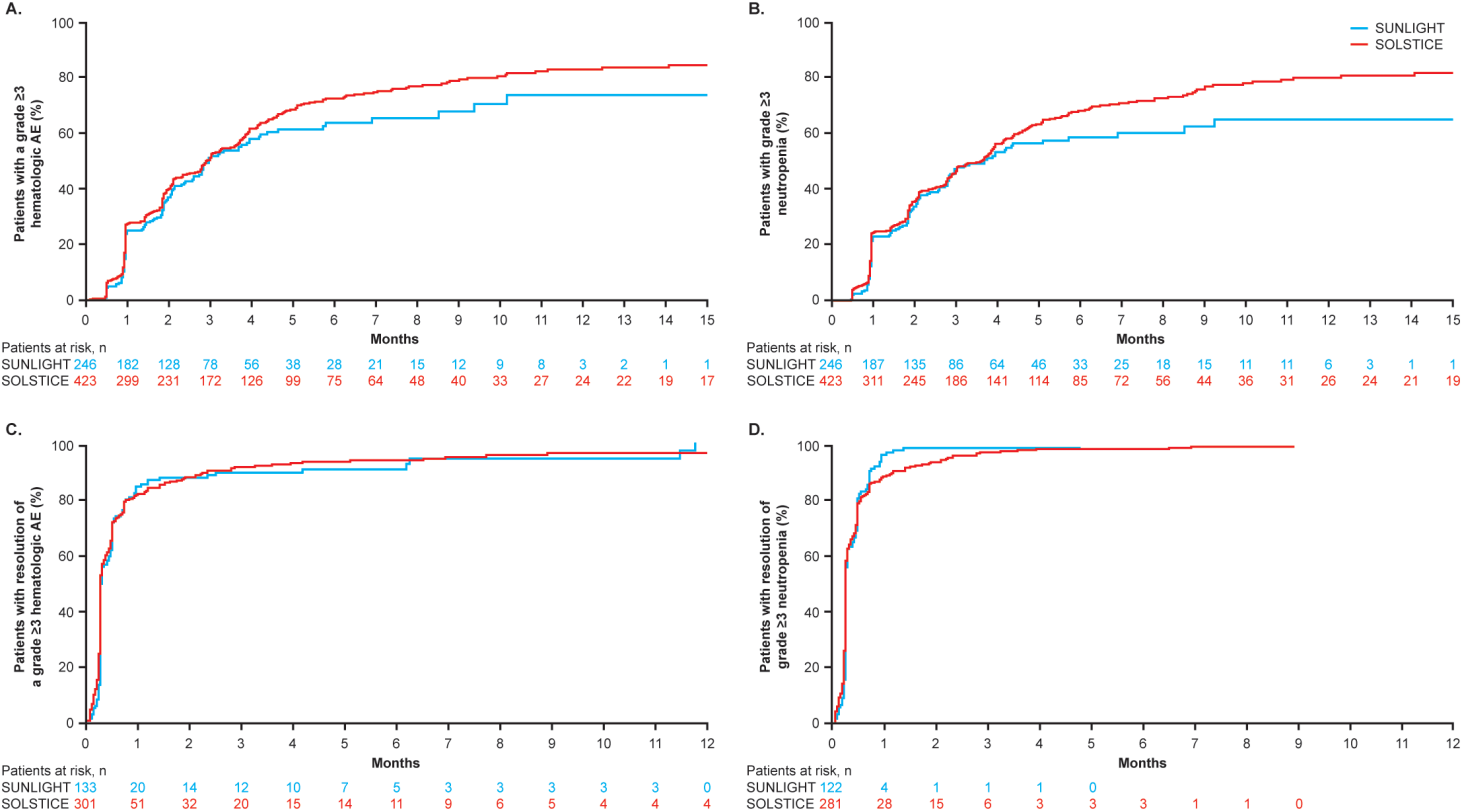
Time to **(A)** onset of grade ≥3 hematologic AEs and **(B)** grade ≥3 neutropenia and to **(C)** resolution of grade ≥3 hematologic AEs and **(D)** grade ≥3 neutropenia to grade ≤2 in the SOLSTICE and SUNLIGHT populations.

In total, 563 (84.2%) patients had at least one hematologic TEAE, including neutropenia (77.0%), anemia (38.7%), and thrombocytopenia (18.4%). Grade ≥3 hematologic TEAEs with onset in cycles 1 and 2 included neutropenia (36.8%), anemia (5.4%), and thrombocytopenia (0.7%). In SOLSTICE and SUNLIGHT, respectively, median time to onset (range) of grade ≥3 hematologic TEAEs was 88 days (3–710) and 90 days (13–309) and that of grade ≥3 neutropenia was 110 days (14–710) and 112 days (14–281) (**Figure 1**). The incidence of any-grade neutropenia was highest in the first four cycles (**Supplementary Table 2**). Grade ≥3 hematologic TEAEs and grade ≥3 neutropenia both resolved to grade ≤2 within a median of 8 days (**Figure 1**).

Overall, 66.5% of patients had a dose modification for neutropenia across the treatment period, most commonly (64.1%) dose delays (**Supplementary Table 3**). In the pooled safety population, 30.6% of patients received at least one concomitant granulocyte colony-stimulating factor (G-CSF) treatment, including 14.5% of patients who received G-CSF within the first two cycles. Median time to use of G-CSF was 34 days in SOLSTICE and 25.5 days in SUNLIGHT. Overall, G-CSF was mostly administered for secondary prophylaxis, and nonpegylated G-CSF was used more frequently than pegylated formulations (28.7% vs. 3.4%).

Hematologic TEAEs resulted in treatment discontinuation in 13 (1.9%) patients overall. There were no fatal hematologic AEs.

### 3.4 Subgroup analyses of grade ≥3 TRAEs

Grade ≥3 TRAEs were more frequent in patients aged ≥75 years than in those aged <75 years, and the frequencies of the most common grade ≥3 TRAEs were generally higher in the older age group (**Table 3**). In the individual SOLSTICE and SUNLIGHT studies and the pooled population, the overall frequencies of grade ≥3 TRAEs tended to be numerically higher in patients with a baseline ECOG PS of 0 compared with those with an ECOG PS of either 1 or 2 individually. There were no clear trends in the frequencies of other common grade ≥3 TRAEs with respect to ECOG PS.

**Table 3:** Most common^a^ grade ≥3 TRAEs by age and ECOG PS.

	SOLSTICE (N = 423)				SUNLIGHT (N = 246)		Total (N = 669)			
Age range	<75 years (n = 235)		≥75 years (n = 188)		<75 years (n = 222)	≥75 years (n = 24)	<75 years (n = 457)		≥75 years (n = 212)	
**Grade ≥3 TRAE, n (%)**	170 (72.3)		159 (84.6)		131 (59.0)	14 (58.3)	301 (65.9)		173 (81.6)	
Neutropenia	146 (62.1)		130 (69.1)		105 (47.3)	13 (54.2)	251 (54.9)		143 (67.5)	
Anemia	18 (7.7)		27 (14.4)		12 (5.4)	0	30 (6.6)		27 (12.7)	
Hypertension	10 (4.3)		12 (6.4)		7 (3.2)	3 (12.5)	17 (3.7)		15 (7.1)	
Diarrhea	12 (5.1)		13 (6.9)		1 (0.5)	0	13 (2.8)		13 (6.1)	
Asthenia	6 (2.6)		11 (5.9)		6 (2.7)	0	12 (2.6)		11 (5.2)	
Leukopenia	12 (5.1)		8 (4.3)		2 (0.9)	1 (4.2)	14 (3.1)		9 (4.2)	
Thrombocytopenia	9 (3.8)		6 (3.2)		4 (1.8)	1 (4.2)	13 (2.8)		7 (3.3)	
ECOG PS	0 (n = 97)	1 (n = 246)		2 (n = 80)	0 (n = 119)	1 (n = 127)	0 (n = 216)	1 (n = 373)		2 (n = 80)
**Grade ≥3 TRAE, n (%)**	82 (84.5)	191 (77.6)		56 (70.0)	76 (63.9)	69 (54.3)	158 (73.1)	260 (69.7)		56 (70.0)
Neutropenia	72 (74.2)	162 (65.9)		42 (52.5)	60 (50.4)	58 (45.7)	132 (61.1)	220 (59.0)		42 (52.5)
Anemia	12 (12.4)	20 (8.1)		13 (16.3)	6 (5.0)	6 (4.7)	18 (8.3)	26 (7.0)		13 (16.3)
Hypertension	4 (4.1)	13 (5.3)		5 (6.3)	6 (5.0)	4 (3.1)	10 (4.6)	17 (4.6)		5 (6.3)
Diarrhea	4 (4.1)	16 (6.5)		5 (6.3)	1 (0.8)	0	5 (2.3)	16 (4.3)		5 (6.3)
Asthenia	2 (2.1)	11 (4.5)		4 (5.0)	3 (2.5)	3 (2.4)	5 (2.3)	14 (3.8)		4 (5.0)
Leukopenia	4 (4.1)	14 (5.7)		2 (2.5)	1 (0.8)	2 (1.6)	5 (2.3)	16 (4.3)		2 (2.5)
Thrombocytopenia	3 (3.1)	9 (3.7)		3 (3.8)	2 (1.7)	3 (2.4)	5 (2.3)	12 (3.2)		3 (3.8)

ECOG PS, Eastern Cooperative Oncology Group performance status; TRAE, treatment-related adverse event.

a Occurring in ≥20 patients in the overall pooled population.

## 4 Discussion

In this pooled safety analysis, FTD/TPI plus bevacizumab demonstrated a predictable and manageable safety profile across all lines of therapy in the continuum of care. Results were consistent across the two phase 3 trials and similar to those reported in phase 2 trials of FTD/TPI plus bevacizumab in first-, second-, and third-line mCRC and several real-world studies (10–12, 16–19). The most common TEAEs were hematologic AEs (e.g., neutropenia and anemia), gastrointestinal toxicities (e.g., nausea and diarrhea), and fatigue. Neutropenia was the most common any-grade/grade ≥3 TEAE in the individual and pooled SOLSTICE/SUNLIGHT populations. Grade ≥3 TEAEs, SAEs, and treatment-related SAEs occurred more frequently in SOLSTICE than in SUNLIGHT, likely reflecting the longer treatment duration in the SOLSTICE study, as well as the fact that, compared with SUNLIGHT, the study population in SOLSTICE tended to be older (median age of 73 vs. 62 years), more frail (19% with an ECOG PS of 2 vs. 0%), and/or had comorbidities that rendered them ineligible for intensive chemotherapy. Additionally, investigators may have been reluctant to enroll patients in SUNLIGHT who had experienced significant toxicity with a previous line of fluoropyrimidine-based therapy.

Any-grade neutropenia was most frequent in the first few treatment cycles and lasted a median of 8 days. Median time to onset of grade ≥3 hematologic AEs was similar in both SOLSTICE and SUNLIGHT, suggesting that prior treatment with chemotherapy and targeted therapy in SUNLIGHT did not predispose patients to earlier-onset neutropenia. Grade ≥3 hematologic AEs, including neutropenia, resolved to grade ≤2 in 8 days, indicating effective management was achieved with dose modifications and/or supportive care interventions, including G-CSF.

Nonpegylated G-CSF was used more commonly than pegylated formulations, and G-CSF was mainly used as secondary prophylaxis, which is consistent with consensus recommendations that primary prophylactic G-CSF use is reserved for chemotherapy regimens with a high (≥20%) risk of febrile neutropenia, and secondary prophylaxis is used in patients who experience febrile neutropenia or dose-limiting neutropenia in a previous treatment cycle (20, 21).

Toxicities considered to be related to bevacizumab occurred less frequently in the SUNLIGHT study than reported in the first-line SOLSTICE study (48.4% vs. 56.5% of patients), potentially reflecting the fact that approximately 72% of patients in the FTD/TPI plus bevacizumab arm had previously received a vascular endothelial growth factor inhibitor before enrollment in SUNLIGHT (4). Among the most frequently reported bevacizumab-related TEAEs was hypertension, which, although usually asymptomatic, can result in cardiovascular complications if unmanaged (22–25). In this pooled analysis, bevacizumab-related hypertension was observed in 8.3% of patients overall, although cardiac events related to bevacizumab treatment were relatively infrequent in SOLSTICE (2.1% in the FTD/TPI plus bevacizumab arm and 1.9% in the capecitabine plus bevacizumab arm) and absent in SUNLIGHT. As the patient population was older in SOLSTICE than in SUNLIGHT, a higher frequency of bevacizumab-related hypertension might have been expected; however, this was found to be similar in both studies. A possible explanation for this could be the slightly higher use of antihypertensives among patients who received FTD/TPI plus bevacizumab in the SOLSTICE trial vs. the SUNLIGHT trial before and during the treatment period (37.3% and 48.1% vs. 30.9% and 37.8%, respectively). In general, the most common grade ≥3 TRAEs occurred more frequently in patients aged ≥75 years than in younger patients, possibly because older patients tend to have more comorbidities, as well as age-associated immune dysfunction that renders them more susceptible to myelosuppression and infections (26). The finding that grade ≥3 TRAEs tended to be more frequent in patients with an ECOG PS of 0 compared with those with a higher score is possibly the result of patients with a lower ECOG PS remaining on treatment for longer, and/or receiving a higher dose intensity of FTD/TPI. A recent retrospective study of FTD/TPI plus bevacizumab as second- or later-line treatment in vulnerable patients with mCRC (median age 79 years)—among whom were several factors associated with intolerance to intensive therapy, including older age (65%), serious concomitant disease (26%), and poor ECOG PS (20%)—found the combination to have an acceptable safety profile (27). Together, the data suggest that that poor functional status may not be predictive of toxicity with FTD/TPI plus bevacizumab, and the combination has acceptable tolerability in vulnerable patients with mCRC.

A limitation of the pooled analysis was that it was not conducted on a matched patient population and there were differences in patient demographics and baseline characteristics between the SUNLIGHT and SOLSTICE trials, including patient age, prior treatment, time on treatment, and ECOG PS. Furthermore, enrolled patients were predominantly White and other ethnicities were underrepresented in both trial populations. In addition, the *post-hoc* nature of the subgroup analyses limits the conclusions that can be drawn from these analyses.

## 5 Conclusions

Overall, the results described herein provide further evidence that FTD/TPI, with or without bevacizumab, is well tolerated. Grade ≥3 hematologic TEAEs were mostly reversible with appropriate management, including dose modifications and prophylactic G-CSF use. The safety profile of the combination was consistent and manageable across first and later lines of treatment, with no new safety concerns.

## Data Availability

Study-level clinical data from this study will be made available upon reasonable request from a qualified medical or scientific professional for the specific purpose laid out in that request and may include deidentified individual participant data. The data for this request will be available after a data access agreement has been signed.
