# Supplementary material for: Pooled safety analysis of two phase 3 studies investigating trifluridine/tipiracil plus bevacizumab in patients with metastatic colorectal cancer
Source: Front Oncol. 2025 Jan 16;14:1506075. doi: 10.3389/fonc.2024.1506075 (PMC11779617; doi:10.3389/fonc.2024.1506075)
Supplement: Supplementary file 1 [file DataSheet1.docx]

# Supplementary Material

*Management of AEs*

In both trials, a new treatment cycle with FTD/TPI could be started only if patients had an absolute neutrophil count of ≥1.5 × 10^9^/L and platelet count of ≥75 × 10^9^/L. Treatment with FTD/TPI was interrupted in the case of absolute neutrophil count <0.5 × 10^9^/L or platelets <50 × 10^9^/L and resumed once counts recovered to the aforementioned starting criteria. If a patient recovered from toxicities requiring treatment interruption (grade 4 neutropenia, grade 4 thrombocytopenia, febrile neutropenia, or grade 3/4 nonhematologic AEs), the FTD/TPI dose could be resumed at a lower dose, reduced in 5-mg/m^2^ decrements to a minimum of 20 mg/m^2^/dose twice daily (Supplementary Table 1). Dose reduction for AEs related to bevacizumab was not recommended. Additionally, treatment with bevacizumab monotherapy was not permitted; therefore, FTD/TPI withdrawal corresponded to treatment withdrawal. If bevacizumab discontinuation was required, patients could continue treatment with FTD/TPI alone.

Neutropenia and anemia were managed with hematologic support (granulocyte colony-stimulating factor [G-CSF], blood transfusions, and erythropoietin-stimulating agents) per institutional site standards (if applicable) or guidelines from the American Society of Clinical Oncology or the European Organisation for the Research and Treatment of Cancer.^1,2^ Recommendations for management of nonhematologic AEs included antiemetics for nausea and vomiting and antidiarrheal therapy for diarrhea.

SUPPLEMENTARY TABLE 1 Recommended dose modifications for hematologic and nonhematologic AEs.^3^

| **Hematologic AEs** |  |
| --- | --- |
| Febrile neutropenia | - Do not start a new treatment cycle until the resumption criteria are met - When resuming dosing, decrease the dose level by 5 mg/m^2^ from the previous dose level - Dose reductions are permitted to a minimum of 20 mg/m^2^/dose twice daily - Do not increase dose after it has been reduced |
| Grade 4 neutropenia (ANC <0.5 × 10^9^/L) that results in more than 1 week’s delay to the start of the next cycle |  |
| Grade 4 thrombocytopenia (platelets <25 × 10^9^/L) that results in more than 1 week’s delay to the start of the next cycle |  |
| **Nonhematologic AEs** |  |
| Grade 3 or 4 nonhematologic AE, except for  grade 3 nausea and/or vomiting controlled by antiemetic therapy or diarrhea responsive to antidiarrheal medicinal products | - Do not start a new treatment cycle until toxicity resolves to grade 1 or baseline - When resuming dosing, decrease the dose level by 5 mg/m^2^ from the previous dose level - Dose reductions are permitted to a minimum of 20 mg/m^2^/dose twice daily - Do not increase dose after it has been reduced |

AE, adverse event; ANC, absolute neutrophil count.

SUPPLEMENTARY TABLE 2 Incidence and duration of neutropenia TEAEs by cycle.

| Cycle | **SOLSTICE**  **(N=423)** | | **SUNLIGHT**  **(N=246)** | | **Total**  **(N=669)** | |
| --- | --- | --- | --- | --- | --- | --- |
|  | Incidence,  n (%) | Median (range) duration, days | Incidence,  n (%) | Median (range) duration, days | Incidence,  n (%) | Median (range) duration, days |
| **All** | 339 (80.1) |  | 176 (71.5) |  | 515 (77.0) |  |
| **1** | 169 (40.0) | 8 (2–626) | 112 (45.5) | 8 (2–146) | 281 (42.0) | 8 (2–626) |
| **2** | 180 (42.6) | 8 (2–271) | 107 (43.5) | 9 (2–370) | 287 (42.9) | 8 (2–370) |
| **3** | 188 (44.4) | 8 (2–394) | 92 (37.4) | 8.5 (4–111) | 280 (41.9) | 8 (2–394) |
| **4** | 180 (42.6) | 8 (2–266) | 71 (28.9) | 11 (3–178) | 251 (37.5) | 9 (2–266) |
| **5** | 160 (37.8) | 8 (2–504) | 46 (18.7) | 9 (2–48) | 206 (30.8) | 8 (2–504) |
| **6** | 140 (33.1) | 9 (2–166) | 34 (13.8) | 8.5 (3–100) | 174 (26.0) | 9 (2–166) |
| **7** | 124 (29.3) | 8 (2–338) | 38 (15.4) | 15 (3–350) | 162 (24.2) | 8 (2–350) |
| **8** | 106 (25.1) | 8 (2–336) | 26 (10.6) | 8.5 (2–57) | 132 (19.7) | 8 (2–336) |
| **9** | 79 (18.7) | 8 (1–136) | 17 (6.9) | 8 (2–57) | 96 (14.3) | 8 (1–136) |
| **10** | 75 (17.7) | 8 (1–127) | 15 (6.1) | 8 (2–36) | 90 (13.5) | 8 (1–127) |
| **11** | 55 (13.0) | 8.5 (1–338) | 10 (4.1) | 8 (2–22) | 65 (9.7) | 8 (1–338) |
| **12** | 46 (10.9) | 8 (3–178) | 14 (5.7) | 9.5 (2–29) | 60 (9.0) | 8 (2–178) |
| **13** | 32 (7.6) | 9 (6–107) | 7 (2.8) | 20 (5–35) | 39 (5.8) | 12 (5–107) |
| **14** | 24 (5.7) | 8 (1–203) | 3 (1.2) | 45.5 (14–77) | 27 (4.0) | 8 (1–203) |
| **15** | 14 (3.3) | 8 (2–28) | 1 (0.4) | 8 (8–8) | 15 (2.2) | 8 (2–28) |
| **16** | 17 (4.0) | 8 (1–63) | 0 | – | 17 (2.5) | 8 (1–63) |
| **17** | 11 (2.6) | 8 (2–29) | 1 (0.4) | 1 (1–1) | 12 (1.8) | 8 (1–29) |
| **18** | 8 (1.9) | 8 (3–9) | 0 | – | 8 (1.2) | 8 (3–9) |
| **19** | 8 (1.9) | 8 (5–22) | 0 | – | 8 (1.2) | 8 (5–22) |
| **20** | 7 (1.7) | 13 (2–49) | 0 | – | 7 (1.0) | 13 (2–49) |
| **21** | 0 | – | 0 | – | 0 | – |
| **22** | 1 (0.2) | 35 (35–35) | 0 | – | 1 (0.1) | 35 (35–35) |
| **23** | 2 (0.5) | 8 (8–8) | 0 | – | 2 (0.3) | 8 (8–8) |
| **24** | 1 (0.2) | – | 0 | – | 1 (0.1) | – |

TEAE, treatment-emergent adverse event.

SUPPLEMENTARY TABLE 3 Dose modifications and G-CSF use in patients with neutropenia, overall and in the first two cycles.

| Patients with neutropenia, n (%) | | **SOLSTICE** | | **SUNLIGHT** | | **Total** | |
| --- | --- | --- | --- | --- | --- | --- | --- |
|  |  | With G-CSF  (N=133) | Without  G-CSF (N=290) | With G-CSF (N=72) | Without  G-CSF (N=174) | With G-CSF (N=205) | Without  G-CSF (N=464) |
| **During the treatment period, neutropenia leading to…** | |  | |  | |  | |
|  | Dose modification | 118 (88.7) | 181 (62.4) | 64 (88.9) | 82 (47.1) | 182 (88.8) | 263 (56.7) |
|  | Any drug interruption | 16 (12.0) | 24 (8.3) | 7 (9.7) | 2 (1.1) | 23 (11.2) | 26 (5.6) |
|  | Dose reduction of any study drug | 23 (17.3) | 19 (6.6) | 5 (6.9) | 6 (3.4) | 28 (13.7) | 25 (5.4) |
|  | Dose delay of any study drug | 108 (81.2) | 176 (60.7) | 63 (87.5) | 82 (47.1) | 171 (83.4) | 258 (55.6) |
|  | Treatment discontinuation | 2 (1.5) | 3 (1.0) | 1 (1.4) | 0 | 3 (1.5) | 3 (0.6) |
| **During the first two cycles, neutropenia leading to…** | |  | |  | |  | |
|  | Dose modification | 82 (61.7) | 114 (39.3) | 53 (73.6) | 66 (37.9) | 135 (65.9) | 180 (38.8) |
|  | Any drug interruption | 4 (3.0) | 2 (0.7) | 2 (2.8) | 2 (1.1) | 6 (2.9) | 4 (0.9) |
|  | Dose reduction of any study drug | 10 (7.5) | 6 (2.1) | 0 | 3 (1.7) | 10 (4.9) | 9 (1.9) |
|  | Dose delay of any study drug | 66 (49.6) | 108 (37.2) | 52 (72.2) | 66 (37.9) | 118 (57.6) | 174 (37.5) |
|  | Treatment discontinuation | 0 | 1 (0.3) | 0 | 0 | 0 | 1 (0.2) |

G-CSF, granulocyte colony-stimulating factor.

# References

1. Smith TJ, Bohlke K, Lyman GH, Carson KR, Crawford J, Cross SJ, et al. Recommendations for the use of WBC growth factors: American Society of Clinical Oncology clinical practice guideline update. *J Clin Oncol*. (2015) 33:3199–212. doi: 10.1200/jco.2015.62.3488
2. Aapro MS, Bohlius J, Cameron DA, Dal Lago L, Donnelly JP, Kearney N, et al. 2010 update of EORTC guidelines for the use of granulocyte-colony stimulating factor to reduce the incidence of chemotherapy-induced febrile neutropenia in adult patients with lymphoproliferative disorders and solid tumours. *Eur J Cancer.* (2011) 47:8–32. doi : 10.1016/j.ejca.2010.10.013
3. LONSURF® (trifluridine and tipiracil) tablets, for oral use [Prescribing information]: Princeton, NJ: Taiho Oncology, Inc; 2023.
